# Interleukin-37: A Link Between COVID-19, Diabetes, and the Black Fungus

**DOI:** 10.3389/fmicb.2021.788741

**Published:** 2022-01-13

**Authors:** Sima Tokajian, Georgi Merhi, Charbel Al Khoury, Georges Nemer

**Affiliations:** ^1^Department of Natural Sciences, School of Arts and Sciences, Lebanese American University, Byblos, Lebanon; ^2^Department of Biochemistry and Molecular Genetics, Faculty of Medicine, American University of Beirut, Beirut, Lebanon; ^3^Division of Genomics and Translational Biomedicine, College of Health and Life Sciences, Hamad Bin Khalifa University, Doha, Qatar

**Keywords:** COVID-19, T2D, corticosteroids, mucormycosis, IL-37

## Abstract

The COVID-19 pandemic involved millions of people and diabetes was identified as an associated comorbidity. Initiation of systemic corticosteroids in patients suffering from severe COVID-19 was associated with lower mortality. A surge of invasive fungal infections of the maxillofacial region, namely mucormycosis, was linked to a deadly infection known as black fungus. Black fungus, diabetes, corticosteroids, and coronavirus disease 2019 (COVID-19) all have a dysregulated immune response in common, which partly could also be attributed to interleukin 37 (IL-37). IL-37, a new cytokine of the IL-1 family, known for broadly reducing innate inflammation as well as acquired immune responses. The use of corticosteroids in diabetic COVID-19 patients, crowded hospitals, and lack of medical oxygen should be carefully considered to reduce COVID-associated secondary infections.

## Graphical Abstract

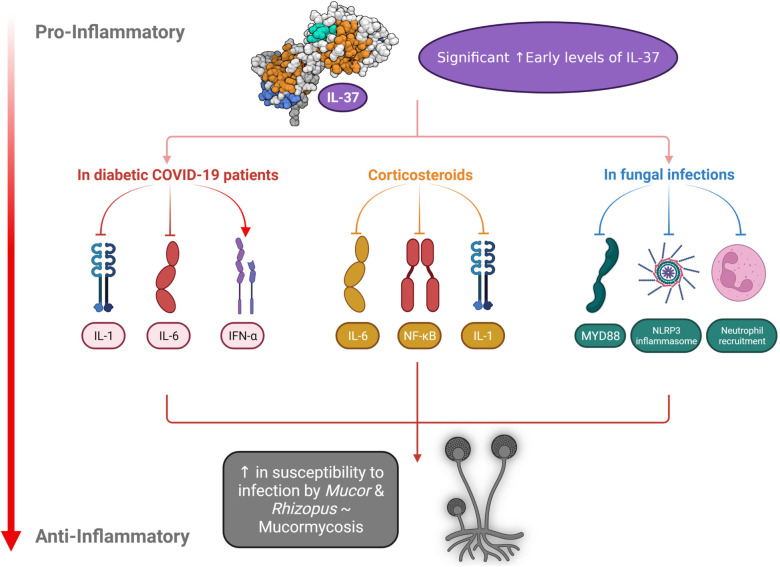

## Introduction

In early December 2019, the first pneumonia cases of unknown origin were identified in Wuhan caused by severe acute respiratory syndrome coronavirus 2 (SARS-CoV-2). The disease is known as coronavirus disease 2019 (COVID-19) with severity ranging from mild to lethal. Symptoms of COVID-19, include, but are not limited to, fever, cough, shortness of breath, muscle aches, anosmia, fatigue, and diarrhea (Molteni et al., 2021). The pandemic involved millions of people with some chronic conditions being associated with severe outcomes. People with preexisting conditions such as diabetes, hyperglycemia, cardiovascular diseases, hypertension, and obesity were linked to higher hospitalization and mortality rates (Rawshani et al., 2018; Corrao et al., 2021). The global diabetes prevalence in 2019 was estimated to be 9.3% (463 million people), with half a billion people suffering (Colagiuri et al., 2019) from diabetes worldwide, and the number was estimated to increase by 25% in 2030. Untreated chronic hyperglycemia imposes damage on numerous cell types and is strongly correlated with limb amputations, kidney failure, and cardiovascular diseases (World Health Organization, 2019). To add a new level of complexity to an already challenging situation, diabetic patients present a high-risk factor for COVID-19 infection. It was pointed out that abnormally high concentrations of glucose in the blood, a feature of diabetes mellitus, may increase the virulence of some pathogens (Joshi et al., 1999; Casqueiro et al., 2012). People with type 2 diabetes (T2D) do not exhibit increased susceptibility to SARS-CoV-2 infection. However, they had more than twice the likelihood for COVID-19-related hospitalization and intensive care admission with a dramatic increase in mortality (Roberts et al., 2020). Guan et al. (2020) showed that 16.2% of the SARS-CoV-2-infected patients that manifested severe symptoms had diabetes. Moreover, results obtained by Zhang et al. (2020) provided further evidence that diabetes was among the most common comorbidities seen (Kayina et al., 2020) among COVID-19 patients. Higher blood glucose levels were also detected in COVID-19 patients. In diabetic this can be attributable to (i) interference of macrophages and T-cell cytokines release diminishing neutrophil recruitment (Delamaire et al., 1997) (ii) reduction of interferon-α (IFN-α) production by blood dendritic cells (Summers et al., 2006) and (iii) disruption of innate immune responses (Zykova et al., 2000). Additionally, the virus surface spike protein facilitates its entry through (Hoffmann et al., 2020) attaching to the human angiotensin-converting enzyme 2 (ACE2) *via* its receptor-binding domain, and targeting ACE2 in the endocrine tissues of the pancreas leading as a result to islets damage (Yang et al., 2010). Liu et al. (2020) revealed the significant pancreatic damage that could occur in patients infected with SARS-CoV-2. The authors expressed mounting concern about the long-term effects of SARS-CoV-2 infection on patients that may be due to its increased infectivity and affinity relative to the other coronaviruses. Here, we review and highlight the interrelationship between T2D and severe clinical course and outcomes of COVID-19 patients.

## Type 2 Diabetes Pathogenesis and the Role Interleukin 37

Obesity leads to adipose tissue dysfunction that was directly linked to the development of health hazards such as diabetes mellitus and cardiovascular diseases. The adipose tissue expansion occurs through hypertrophy (increase in existing adipocyte size) and hyperplasia (increase in adipocyte number) (Greenberg and Obin, 2006). The fat tissue was recognized as a highly active endocrine organ mainly producing inflammatory mediators known as adipokines (Kershaw and Flier, 2004). Adipokines are a family of hormones having anti- or pro-inflammatory effects (Tilg and Moschen, 2006). In the event of obesity, more pro-inflammatory cytokines were produced when compared to the anti-inflammatory ones (Shibata et al., 2017). For that, T2D is associated with low-grade chronic inflammation linked to excessive visceral adipose tissue, affecting homeostatic glucose regulation and peripheral insulin sensitivity (Iacobellis, 2020). Tumor necrosis factor α (TNF-α), resistin, and interleukin-6 (IL-6) were linked previously to insulin resistance, while adiponectin was found to enhance insulin sensitivity, increase glucose transepithelial transport, lower glucose synthesis in the liver, and suppress IL-6 and TNF-α (Yamauchi and Kadowaki, 2008).

**FIGURE 1:**
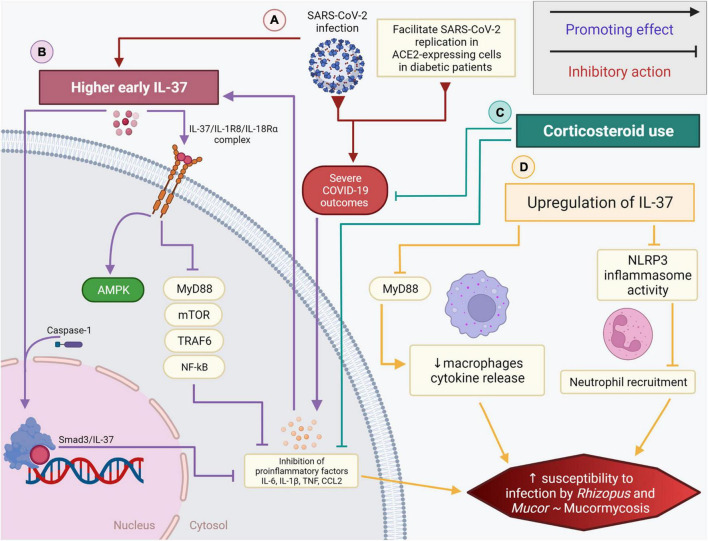
The role of IL-37 and corticosteroids in diabetic COVID-19 patients and their association with the black fungus infection. **(A)** SARS-CoV-2 infection in hyperglycemic patients promotes a dysregulated pro-inflammatory cytokine storm while also promoting higher early expression of IL-37 **(B)** IL-37/IL-1R8/IL-18Rα complex inhibits the mobilization of MyD88, mTOR, TRAF6, NF-κB, subsequently impeding the secretion of pro-inflammatory cytokines. IL-37 could be directly processed by caspase-1, intracellularly, and associates with SMAD3 in the nucleus. **(C)** Corticosteroids and the loss of immune homeostasis linked partly to IL-37, coupled with diabetes, can increase the risk of black fungus infection/mucormycosis. **(D)** Higher early IL-37 and corticosteroid use in diabetic COVID-19 patients inhibit proinflammatory signals. The upregulation of IL-37 interferes with the innate immune system driven inflammation by promoting the decrease of macrophage cytokine release, reducing the NLRP3 inflammasome activity and inhibiting neutrophil recruitment. These factors, when combined, significantly elevate the risk and susceptibility to mucormycosis.

Inflammation has an active role in the pathogenesis of hyperglycemia (Gianchandani et al., 2020), and acute viral respiratory infection by SARS-CoV was linked to transient insulin resistance and hyperglycemia induced by a temporary inflammation of the pancreas’ islet cells (Yang et al., 2010). Stress hyperglycemia, which is a temporary increase in blood sugar, may have a negative impact in acute illness (Corrao et al., 2021). Chronic hyperglycemia and inflammation can cause an abnormal and ineffective immune response with the combined elevated levels of interleukin-1β (IL-1β), IL-6, and C-reactive protein (CRP) being predictive of T2D (Manson et al., 2001; Figure 1). Hypoxia and cell death of expanding adipose tissue, activation of the nuclear factor-κB (NF-κB) and JUN *N*-terminal kinase (JNK) pathway, activation of interleukin-1β (IL-1β), and recruitment and activation of immune cells were all linked to the inflammatory state in T2D (Donath and Shoelson, 2011; Gianchandani et al., 2020). IL-1 family cytokines play a crucial role in immune homeostasis. Strikingly, most proinflammatory factors present at high levels in the blood of patients with T2D were IL-1 dependent, with IL-37 adjusting obesity-induced inflammation and insulin resistance (Ballak et al., 2014; Table 1). IL-37 plays an important role in metabolic and autoimmune diseases as well as infections. The development in the *in silico* research of human databases has led to the discovery of IL-37 by several independent groups (Boraschi et al., 2011). Fragments of mRNA sequence encoding an IL-1-like molecule were expressed in human databases (accession number AI014548) and characterized by having five different isoforms (IL-37a-e) (Busfield et al., 2000; Kumar et al., 2000; Pan et al., 2001). IL-37, which can be produced in intra- and extracellular domains, was linked to anti-inflammatory actions through inhibiting innate responses (Dinarello and Bufler, 2013) and detected in the nucleus affecting gene transcription. The expression levels of TNF, IL-1, IL-6, and chemokine MIP-2 were significantly decreased in lipopolysaccharide (LPS)-stimulated macrophages transfected with human IL-37 when compared to those transfected with an empty plasmid (Nold et al., 2010). Sharma et al. (2008) showed that efficient caspase-1 inhibitors can reduce IL-37 entry to the nucleus, and hence caspase-1 processing could be fundamental for IL-37 translocation. Translocation to the nucleus, after caspase-1 processing, involves SMAD3, which is part of TGF-β anti-inflammatory signaling pathway (Figure 1). Investigations were conducted to confirm the functional interaction of SMAD3 with IL-37. Nold et al. (2010) observed the formation of IL-37-SMAD3 complex in perinuclear and cytosolic regions in IL-37-transfacted A549 cells. This correlated favorably with the results obtained previously (Bar et al., 2004) and which demonstrated that the inhibition of SMAD3 could be accountable in reversing the inhibition of IL-6 and IL-1 expression in cells transfected with IL-37. Furthermore, it was exhibited that the potential for IL-37 to reduce the production of IL-1, IL-9, IL-6 and TNF decreased after introducing short hairpin RNA (shRNA) into human macrophages and which inhibited SMAD3 expression (Nold et al., 2010). IL-37 exerts inhibitory properties on the innate inflammatory and acquired immune responses and has a dualfunction with both intra- and extracellular mechanisms of action (Bulau et al., 2014). IL-18 induces activation of IL-1 receptor-associated kinase and tumor necrosis factor (TNF) receptor-associated factor 6 (TRAF-6), with the net outcome of activating the kinases being the translocation of NF-κB (Matsumoto et al., 1997). IL-37 may also inhibit IL-18 dependent pro-inflammatory cytokine production (Ballak et al., 2014). IL-37b and IL-18 are phylogenetically related, and both have two symmetric key structural amino acid residues (Glu-35 and Lys-124) (Dinarello and Bufler, 2013). IL-37 could also interact and bind, but with low affinity, to IL-18 receptor α subunit (IL-18Rα) (Dinarello and Bufler, 2013). IL-37, however, binds to the IL-18 binding protein (IL-18BP), the natural inhibitor of IL-18, and forms a complex reducing IL-18’s activity. IL-37b is the receptor antagonist of IL-18, it binds to the IL-18 binding protein (IL-18BP) followed by binding to the IL-18Rα, recruiting IL-1R8, and forming the IL-37/IL-1R8/IL-18Rα complex (Donath and Shoelson, 2011).

**TABLE 1:** The role of IL-37 and its biological functions in infectious and metabolic syndrome associated diseases.

Biological function/Role of IL-37 in infectious diseases and metabolic syndrome and associated diseases	Mechanism of function	References
Protective against Gram-negative LPS induced septic shock	Downregulating the expression of proinflammatory cytokines IL-6, INF-γ, IL-1β, IL-17 and attenuating the action of DCs.	Nold et al., 2010
Inhibits HIV replication	Significant increase in IL-37 mRNA transcription.	Su and Tao, 2021
Potent anti-inflammatory agent in viral-mediated myocarditis	Lowers IL-6 and IL-17A levels. Increases IL-10 expression. Modulates Th17 and Treg cells functions.	An et al., 2017
Linked to significant increase in liver tissue damage in chronic Hepatitis B patients	Not well understood	Li et al., 2013
Higher early IL-37 responses linked to positive COVID-19 prognosis	Blocking IL-1 related cytokine storm. Lowers levels of IL-6 and IL-8. Increases levels of IFN-α.	Su and Tao, 2021
Disseminated candidiasis	Inhibits TNF-α expression and neutrophil recruitment.	van de Veerdonk et al., 2015
Limits inflammatory response mediated by *Aspergillus* infection- protective for lung tissues.	Lowers NLRP3 dependent neutrophil recruitment and inflammasome activity. Decrease in IL-1β secretion.	Allam et al., 2020
Poor prognosis of Eumycetoma which is driven by infection with *Madurella mycetomatis*.	Diminished levels of IL-1β.	Abushouk et al., 2019
Reduces pro-inflammatory cytokine secretion in adipose tissue ameliorating metabolic syndrome associated diseases- obesity and type 2 diabetes (T2D)	Reduction in lipid buildup in the liver Boosts insulin-dependent response and decreases macrophage permeability to adipose tissue Increase in the release of adiponectin coupled with the decrease of leptin in adipose tissue	Cavalli and Dinarello, 2018, Su and Tao, 2021
Athero-protective in atherogenesis Sustains plaque stability in COVID-19 patients	Lessens oxidized low-density lipoprotein (oxLDL) buildup. Downregulates Treg activation, with a reduction in IL-1β, IL-6 and IL-12 expression.	Law et al., 2021

## Correlation Between Interleukin 37 and COVID-19

The potential involvement of IL-37 in suppressing the cytokine storm that contributes to poor prognosis and mortality in COVID-19 patients was studied (Ragab et al., 2020). The mass release of pro-inflammatory cytokines causes acute respiratory distress syndrome and multiple organ failure (Ragab et al., 2020). Controlling the cytokine storm is critical in preventing the progression of the disease and saving patients’ lives. A noteworthy strategy that comes into play are anti-inflammatory factors, such as IL-37, that can maintain biochemical homeostasis. Since IL-37 is considered as the natural inhibitor of IL-1, the main modulator of pro-inflammatory cytokines and inflammatory responses, it is safe to assume that IL-37 could provide relief in systemic inflammation that occurs after COVID-19 infection (Table 1). Recently, the correlation between IL-37 production, pro-inflammatory cytokines level, and clinical outcomes in SARS-CoV-2 infection was revealed (Li et al., 2021). An enzyme linked immunosorbent assay demonstrated significantly higher concentrations of IL-37 in the plasma of SARS-CoV-2-infected subjects when compared to healthy ones. Li et al. (2021), showed that mild and severe clinical outcomes are directly associated with reduced and elevated plasma levels of IL-37, respectively. Given the fact that high early IL-37 responses were accompanied with speedy recovery among hospitalized patients (earlier viral clearance, chest tomographic improvement, and cough relief) (Table 1), the authors suggested that IL-37 administration may be used to maintain a balanced inflammatory response (Li et al., 2021). Moreover, Ahmed and Ad’hiah (2021a) revealed the role of IL-37 in the virus-mediated pathogenesis. In a case-control study involving 200 patients, the sequencing of the *IL37* gene (Gene ID: 27178) identified a couple of single nucleotide polymorphisms (SNPs) that were directly linked with the susceptibility to COVID-19. The two variants of *IL*37 gene with rs3811046 and rs3811047 SNPs, may increase the risk of developing COVID-19 by 2.55 and 2.02-fold. The authors showed a significant correlation between the presence of these polymorphisms and increased incidence of severe COVID-19. The data revealed that SNP rs3811046 detection was significantly higher in patients (61%) when compared to controls (38.0%), which was also the case with SNP rs3811047 (patients 39% vs. controls 24%). In contrast to the previous findings, a more recent study showed that IL-37 expression was suppressed in COVID-19 patients but was prominent in obese patients (Ahmed and Ad’hiah, 2021b). Therefore, it is evident that low IL-37 plasma levels could be used as COVID-19 severity marker. IL-37 was also found to ameliorate influenza pneumonia *in vivo* (Qi et al., 2019). Interestingly, treatment of H1N1-infected mice using IL-37 reduced (by 2 days) the body weight recovery time. The body weights of mice treated with oseltamivir phosphate in combination with IL-37 and oseltamivir phosphate alone began to increase at 7- and 9-days post-inoculation, respectively. The intravenous IL-37 administration improved the survival rates in H1N1- infected mice with a total of 71% of the mice surviving, compared to 57% survival when using a single dose of oseltamivir phosphate. IL-37 additionally relieved H1N1-induced lung tissue damage. The correlation between IL-37 and severity of viral infection was noteworthy. IL-37 treatment modulates (suppresses) the immune response by shifting the cytokine equilibrium away from excess inflammation. Qi et al. (2019) also demonstrated that IL-37 can reduce the levels of pro-inflammatory modulators in mice infected with respiratory viruses. Using RT-qPCR as well as Cytometric Beads Array, they showed that IL-37 treatment significantly reduced the excessive production of IL-6, TNF-α, MCP-1, IL-1α, IL-1β, MIP-1α, MIP-1β, IP-10, MIG, RANTES, IFN-γ and IL-10 in the lung tissues of infected mice.

## Diabetes and COVID-19

Diabetes has been identified as comorbidity associated with COVID-19 severity. Higher risks of COVID-19-related mortality was linked to cardiovascular and renal complications associated with diabetes, glycemic control, and body mass index (BMI) (Holman et al., 2020). Blood glucose level was revealed to be a key mediator of severe symptoms in COVID-19. Persistent inflammation is associated with a compensatory anti-inflammatory response. T2D-related immunosuppression may facilitate SARS-CoV-2 replication in ACE2-expressing cells, promoting cytokine storms and inducing immune dysregulation, partly explaining the more severe COVID-19 outcomes in patients with diabetes (Roberts et al., 2020). IL-37 performs its immunosuppressive activity by acting on mTOR and increasing the adenosine monophosphate (AMP) kinase. It inhibits class II histocompatibility complex (MHC) molecules and inflammation in inflammatory diseases by suppressing MyD88 and subsequently IL-1β, IL-6, TNF, and CCL2 (Conti et al., 2020; Figure 1). Li et al. (2019) revealed the association between IL-37 and T2D. The authors showed an overexpression of IL-37 in the peripheral blood monocyte cells of T2D patients when compared to healthy controls. A significant difference was detected using ELISA between the serum level of IL-37 in T2D vs. control subjects. Additionally, the study showed that gut microbiota dysbiosis significantly contributed to the development of T2D and that a better response to insulin therapy was seen in patients with higher IL-37 levels (Ballak et al., 2014; Li et al., 2019). Furthermore, a mouse model receiving high-fat diet treatment provided further evidence on the link between IL-37 and T2D. IL-37 increases insulin sensitivity and reduces gut microbiota dysbiosis.

## Corticosteroids and the Black Fungus

Numerous therapeutic options to modulate the inflammatory response against SARS-CoV-2-infection were considered. It is widely known that corticosteroids administration successfully prohibits extensive immune responses and accordingly used in controlling hyperinflammation and acute respiratory distress syndrome (Barnes, 2006). During the COVID-19 pandemic, an alarmingly high prevalence of self-medication was reported (Quispe-Cañari et al., 2021), with the corticosteroids being one of the most drugs used due to their high affordability and accessibility (Chaudhry et al., 2021). The RECOVERY Collaborative Group demonstrated that the incidence of death was lower in patients treated with dexamethasone (29.3%) when compared to those who received usual care (41.4%) (Recovery Collaborative Group, 2021). The World Health Organization (WHO) has recommended against using corticosteroids for COVID-19 treatment, but this recommendation was later reversed (World Health Organization, 2020). Corticosteroids regulate inflammation and can block the function of transcription factors mediating inflammatory responses, such as NF-κB and activator protein 1 (AP-1) and inhibit the production of proinflammatory cytokines (IL-1, TNFα, IL-6) (Cain and Cidlowski, 2017). However, the utilization of corticosteroids appears to be a double-edged sword against COVID-19. Their use in respiratory viral infection was linked to an increased risk of secondary opportunistic bacterial or fungal infections (Conti et al., 2020). A surge of invasive fungal infections (IFI) of the maxillofacial region, namely mucormycosis, was reported in India and linked to a rare and possibly deadly infection known as black fungus. Mucormycosis, previously known as zygomycosis, is rare, though increasingly prevalent, and a dangerous angio-invasive infection caused by mucormycetes (Skiada et al., 2020). The most common causative agents of the disease are *Rhizopus* and *Mucor* (Roden et al., 2005). These ubiquitous fungi can be found particularly in the soil in association with decaying leaves, compost piles, and rotten wood (Sridhara et al., 2005). Mucormycetes infections occur more frequently in summer and fall compared to the winter and spring and affect the sinuses and the lungs after inhaling fungal conidia (Sugar, 1990). The fungus can also be introduced through a break in the skin such as a cut, burn, or other types of skin injury (Roden et al., 2005). The mucormycetes invade blood vessels which may reduce the blood and nutrient flow to downstream organs consequently leading to necrosis (Skiada et al., 2020). Hosts susceptible to mucormycosis include diabetics, those on systemic corticosteroid use, and immunocompromised individuals. Healthcare-associated mucormycosis is a matter of concern and was previously linked to the use of contaminated devices or environmental reservoirs (Rammaert et al., 2012b).

Opportunistic fungal infections, such as the black fungus commonly seen in diabetics, hyperglycemia, COVID-19 patients, and linked to corticosteroids have all in common a dysregulated immune response that partly could also be attributed to IL-37 (Rammaert et al., 2012a; van Paassen et al., 2020; Kumar et al., 2021). A significant correlation exists between well-controlled blood glucose and lower serum levels of inflammatory markers such as IL-6, high sensitivity c-reactive protein (CRP), and lactate dehydrogenase in COVID-19 patients (Gianchandani et al., 2020). Corticosteroids, on the other hand, can downregulate the expression of IL-37, which plays an essential regulatory role in innate and adaptive immunity. Its expression in human adipose tissue was associated with enhanced insulin sensitivity and reduced adipose tissue inflammation (Table 1; Ballak et al., 2014). IL-37 exerts significant anti-inflammatory and metaboregulatory effects, dramatically reducing cytokine secretion in macrophages and dendritic cells, with higher early IL-37 responses in COVID-19 patients leading to earlier viral RNA negative conversion (Wang et al., 2018; Yi et al., 2020). It protects the body by inhibiting excessive immune response. Still, it could also lead to increased infection and mortality caused by some pathogens, possibly due to its inhibitory effects on the immune response. The regulatory role it plays could explain the link between the severity of COVID-19 in some diabetic patients being treated with corticosteroids and their potential to additionally acquire black fungus disease in poorly maintained critical care units.

## Interleukin 37 and Its Central Role in COVID-19, Diabetes, and Mucormycosis

Most serious fungal disease arises from poorly cleared infection or disrupted barrier integrity (Rautemaa-Richardson and Richardson, 2021). Mucormycosis is an invasive and potentially life-threatening opportunistic fungal infection. The predisposing conditions vary geographically with diabetes mellitus being the most common risk factor in Asia (Hoang et al., 2020). Different subtypes of monocytes and macrophages play a pivotal role in the production of pro-inflammatory mediators and initiation of an inflammatory response. Previously Waldorf et al. (1984) showed that bronchoalveolar macrophages formed part of an efficient defense against *Rhizopus oryzae* by inhibiting germination and interfering with the conversion of *R. oryzae* to its tissue invasive phase. Neutrophils and monocytes are critical components of the host response to mucormycosis which efficiently eliminate fungal spores and hyphae by oxidative and non-oxidative killing mechanisms (Diamond et al., 1982; Waldorf et al., 1984). Enhanced susceptibility to mucormycosis was observed in human diabetics. Hyperglycemia and acidosis are known to impair chemotaxis and the killing activity of phagocytic cells against Mucorales by weakening oxidative and non-oxidative mechanisms (Chinn and Diamond, 1982). Corticosteroids, on the other hand, act on monocytes and macrophages, induce anti-inflammatory mediators and differentiation of anti-inflammatory phenotypes, and limit T-cell activation (Ehrchen et al., 2019).

IL-37, as mentioned above, is also an inhibitor of innate immunity through the caspase-1-dependent nuclear translocation or by the extracellular mechanism engaging the IL-18 receptor (Figure 1 and Table 1; Bulau et al., 2014). The timing of IL-37 expression at the site of inflammation is critical. IL-37 based anti-inflammatory strategies could have a negative impact on susceptibility to infections by inhibiting host defenses or be beneficial for the outcome of the host. Higher early IL-37 responses were correlated with earlier viral RNA negative conversion, chest computed tomographic improvement, and cough relief in COVID-19 patients (Li et al., 2021). In contrast, IL-37 interfered with the innate protective anti-*Candida* host response by reducing the production of proinflammatory cytokines and suppressing neutrophil recruitment in response to *Candida* (Table 1), resulting in an increased susceptibility to disseminated candidiasis (van de Veerdonk et al., 2015).

## Conclusion

There are several measures that can be employed to minimize COVID-19 mortalities and reduce the risk of mucormycosis in people with diabetes. Maintaining and monitoring the glucose level is essential to improve outcomes and reduce secondary infections, renal dysfunction, ICU stay, and ventilator dependence. The pro-inflammatory metabolic state in diabetic patients could promote a virus-linked inflammatory surge leading to insulin resistance and severe hyperglycemia. People with diabetes and COVID-19 should be monitored for inflammatory and prothrombotic markers (Gianchandani et al., 2020). CRP is a good inflammatory marker revealing the association between diabetes and adverse COVID-19 outcomes, including the need for ICU admission, dyspnea, and length of stay (Koh et al., 2021), whereas fibrinogen for coagulation dysfunction (Varikasuvu et al., 2021). The use of corticosteroids can modulate the risk when used prudently. Limitations to their use associated with corticosteroid-mediated inhibition of glucose uptake and reductions in insulin sensitivity in COVID-19 patients and their role in increasing the susceptibility to acquire other infections are among the major concerns. IL-37 is a cytokine that exerts anti-inflammatory effects both intracellularly and extracellularly and is a therapeutic approach that should be considered and further studied. Finally, epidemiological observations combined with molecular epidemiology and experimental data are needed to understand and uncover risk factors related to the host, the pathogen, and variants of concern.

## Author Contributions

ST: conceptualization. ST, GM, CA, and GN: investigation, writing—original draft, and writing—review and editing. ST and GN: validation. All authors contributed to the article and approved the submitted version.

## Conflict of Interest

The authors declare that the research was conducted in the absence of any commercial or financial relationships that could be construed as a potential conflict of interest.

## Publisher’s Note

All claims expressed in this article are solely those of the authors and do not necessarily represent those of their affiliated organizations, or those of the publisher, the editors and the reviewers. Any product that may be evaluated in this article, or claim that may be made by its manufacturer, is not guaranteed or endorsed by the publisher.
